# Designing an Agent-Based Model for Childhood Obesity Interventions: A Case Study of ChildObesity180

**DOI:** 10.5888/pcd13.150414

**Published:** 2016-01-07

**Authors:** Erin Hennessy, Joseph T. Ornstein, Christina D. Economos, Julia Bloom Herzog, Vanessa Lynskey, Edward Coffield, Ross A. Hammond

**Affiliations:** Author Affiliations: Joseph T. Ornstein, Department of Political Science, University of Michigan, Ann Arbor, Michigan; Christina D. Economos, Julia Bloom Herzog, Vanessa Lynskey, ChildObesity180, Friedman School of Nutrition Science and Policy, Tufts University, Boston, Massachusetts; Edward Coffield, Department of Health Professions, Hofstra University, Hempstead, New York; Ross A. Hammond, Center on Social Dynamics and Policy, The Brookings Institution, Washington, DC.

## Abstract

Complex systems modeling can provide useful insights when designing and anticipating the impact of public health interventions. We developed an agent-based, or individual-based, computation model (ABM) to aid in evaluating and refining implementation of behavior change interventions designed to increase physical activity and healthy eating and reduce unnecessary weight gain among school-aged children. The potential benefits of applying an ABM approach include estimating outcomes despite data gaps, anticipating impact among different populations or scenarios, and exploring how to expand or modify an intervention. The practical challenges inherent in implementing such an approach include data resources, data availability, and the skills and knowledge of ABM among the public health obesity intervention community. The aim of this article was to provide a step-by-step guide on how to develop an ABM to evaluate multifaceted interventions on childhood obesity prevention in multiple settings. We used data from 2 obesity prevention initiatives and public-use resources. The details and goals of the interventions, overview of the model design process, and generalizability of this approach for future interventions is discussed.

## Background

Researchers have called for the development of systems-science models to study topics in public health (1–6) and evaluate interventions (7,8). Such models have made advances in infectious disease epidemiology (9,10) but have not been broadly used for studying chronic disease prevention. Agent-based modeling (ABM) is a promising computational tool for research on childhood obesity prevention (6). “Agents” are defined by the users: they can be cellular organisms, people, companies, cities, products, and so forth. In some fields, ABM is referred to as “individual-based” modeling. The approach is especially useful when detailed information about individual behavior exists, but the influence of an intervention on that behavior is uncertain (1). An ABM starts by defining properties and behaviors of agents and then simulates how agents change and interact over time. Using ABMs to assess how heterogeneous agents respond to changes in their environment provides a laboratory to examine outcomes while accounting for pathways through which an intervention may influence behavioral change.

Despite the potential of ABMs in examining how people may react to an intervention, few articles have discussed the steps necessary to create such models. This article aims to address this gap. Using a case study approach, it explains the procedures followed and the challenges arising in the process of designing an ABM to inform interventions for preventing childhood obesity. The emphasis of this article is on process rather than findings — modeling results or empirical tests of the prototype model are not reported. The Tufts University Institutional Review Board approved the interventions described in this article.

## Case Study

ChildObesity180, based at Tufts University, is an organization committed to reversing the trend of childhood obesity (www.childobesity180.org). The organization combines research and evaluation, innovative strategies, multisector collaboration, and eventual widespread promulgation of practices. The organization’s goal is to use an evidence-based approach to develop initiatives with long-term impact and to monitor and measure performance of the initiatives against established goals. ChildObesity180 consists of 4 initial areas of intervention targeting school-aged children: healthy school breakfast, quality physical activity in schools, healthy eating and active time during out-of-school programming, and healthier eating in restaurants. This article uses data from 2 ChildObesity180 interventions: Active Schools Acceleration Project (ASAP) and Healthy Kids Out of School (HKOS). ASAP (www.activeschoolsasap.org) aims to increase daily moderate-to-vigorous physical activity (MVPA) through 3 different school-based physical activity programs. Program 1, a before-school program; Program 2, a classroom-based program; and Program 3, a walking/running program offered throughout the school day. The goal of HKOS (www.healthykidshub.org/) is to create healthy out-of-school-time (OST) programs in various environments. OST programs adopt and implement 3 evidence-based principles: 1) Drink Right: choose water instead of sugar-sweetened beverages; 2) Move More: boost movement and physical activity in all programs; and 3) Snack Smart: fuel up on fruits and vegetables (10). 

## Steps to Design an Agent-Based Model

One challenge in designing an ABM is to balance realism and parsimony (11). A model that is too simple may fail to offer insight into the particular problem, whereas one that is too complex will be difficult to understand and interpret. Deciding how much detail to include for any given problem is critical. A first step for any modeling effort is to specify the goals and questions for the model. This decision guides implementation choices.

The goals of our ABM were to 1) develop a mechanistic model of the baseline context into which ChildObesity180 interventions are introduced, as well as how each intervention functions (to improve realism of impact estimates); 2) anticipate how each intervention might work across diverse populations; and 3) provide a computer platform to explore how the interventions could grow or be modified to maximize impact. The questions driving the model’s construction were the following: 1) how childhood body mass index (BMI) changes over time and 2) how this change is affected by decreased caloric intake or increased physical activity as a result of the interventions. These questions drove each decision to include or exclude a variable in the model design. Constructing and testing the ABM followed a 5-step process.

### Step 1. Characterize BMI dynamics of the simulated agents

ABM requires the modeler to define the properties of each agent and specify how the properties change over time. Two questions must be answered for each property: 1) what the initial distributions are across the population and 2) what rules determine change over time. For example, for our model, the outcome was change in body mass index (BMI), and it has the properties of height and weight. Agents in the model grow in height at an empirically derived rate (estimated using the Centers for Disease Control and Prevention’s [CDC’s] growth charts [12]) uncoupled from behavior. The average rate of growth is conditional on age and sex, which required the modeling of age and sex as agent properties. These properties were assigned to agents based on empirical distributions informed by US Census data (13): 51% boys with a uniform distribution over ages 6 to 12. Sex does not change, but age increases linearly with time.

Multiple methodologies exist to specify body weight dynamics (14–16). For this study, the ABM required a model of weight change that 1) would apply to children aged 6 to 12, and 2) would require just enough inputs to model reality while adhering to the ABM parsimony design element. Based on these criteria, a hybrid model was built that translated caloric intake, time spent in physical activity, and physical activity intensity into changes in BMI. The hybrid model computed energy balance (calorie intake minus calorie expenditure) and assumed that a calorie surplus or deficit translates into weight change at a rate of 7,700 calories per kilogram.

Two final factors required initial distributions and trajectories before the BMI change property could be fully characterized: calorie expenditure and calorie intake. Calorie expenditure was modeled using the standard Schofield equation (14), which estimates resting metabolic rate (RMR) from height (*H*), weight (*W*), age, and sex:

For boys aged 3 to 10 years, RMR = 19.59*W* + 1.303*H* + 414.9

For boys aged 10 to 18 years, RMR = 16.25*W* + 1.372*H* + 515.5

For girls aged 3 to 10 years, RMR = 16.969*W* + 1.618*H* + 371.2

For girls aged 10 to 18 years, RMR = 8.365*W* + 4.65*H* + 200.0

Calorie expenditure, in turn, was computed as the product of RMR from the Schofield equations and a multiplier signifying the duration and intensity of daily physical activity.

**Figure 1:**
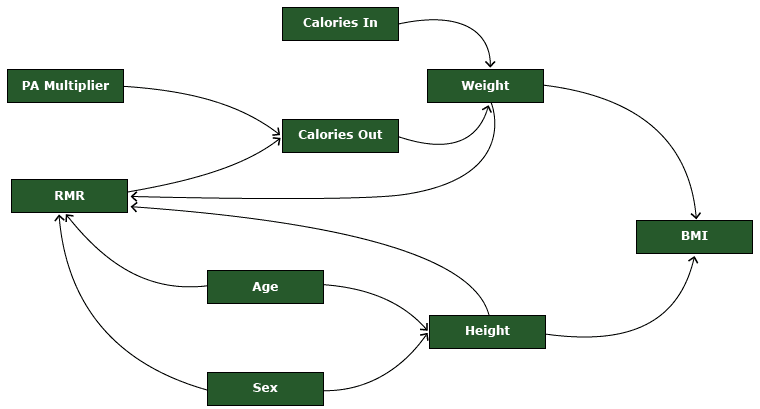
A visual representation of the agent-based model describing BMI dynamics in each agent. Abbreviations: BMI, body mass index; PA, physical activity; RMR, resting metabolic rate.

Daily calorie intake was estimated using National Health and Nutrition Examination Survey (NHANES) 2003–2006 data (17,18); when we designed our model, there were no models that predicted food consumption for children aged 6 to 12 years. Using NHANES data and controlling for anthropometric and demographic data, we regressed calorie intake on RMR. The resulting regression coefficient defined children’s daily calorie intake without the intervention. This assumption provided a baseline estimate from which the effects of interventions could be estimated while also computing realistic growth patterns of the simulated children (Figure 1). 

### Step 2. Characterize the environments in which agents live

To move from the individual-level model of BMI change to a population-level model of BMI change, each agent had to be assigned an initial value for each key variable. Each assignment was drawn from an empirical distribution.

Another important tension in the design of an ABM is the balance between empirical fidelity to one context (for example, settings in which ChildObesity180 is active and collecting data) and generalizability. Balancing these goals requires close attention to the stated design and questions. Because the central purpose of our model was to assist ChildObesity180 in considering expansion and modification of the interventions, an effort was made to preserve generalizability by creating stylized settings (in other words, “town types”) based on population-based survey data.

For example, weight and physical activity level vary according to context. Our model defined 3 town types: town A (average childhood obesity rates), town B (higher than average childhood obesity rates), and town C (lower than average childhood obesity rates). In each town type, the initial BMI distribution was generated using the LMS method (12). The LMS method transforms a standard normal random variable into a skewed normal random variable, with given median (*M*), standard deviation (*S*), and power in the Box–Cox transformation (*L*). The *S* and *L* parameters are drawn from the CDC’s growth charts, which are conditional on age and sex; *M *parameter varies based on town type (Table).

**Table:** An Agent-Based Model^a^ Designed for Two Childhood Obesity Prevention Interventions^b^: Properties and Data Inputs by Stylized Town Type^c^

Characteristic	Stylized Town Type		
	Town A — Average Childhood Obesity Rates	Town B — Higher Than Average Childhood Obesity Rates	Town C — Lower Than Average Childhood Obesity Rates
**Town**			
General	Empirically derived US population average from NHANES 2003–2006 data	Has a lower socioeconomic status, a larger proportion of racial/ethnic minority residents, and a higher obesity rate than town A	Has a higher socioeconomic status, a smaller proportion of racial/ethnic minority residents, and a lower obesity rate than town A
Sex	51.1% boys, 48.9% girls; based on empirical distributions informed by US Census data (13) for children aged 6–12 y	Same as town A	Same as town A
Race/ethnicity	Estimates generated from NHANES 2003–2006 data	Higher than town A	Lower than town A
Socioeconomic status	Qualitatively assigned as Middle	Qualitatively assigned as Low	Qualitatively assigned as Upper
**Agent**			
Age	Uniform, random distribution of children aged 6–12 y	Same as town A	Same as town A
Sex	Random distribution	Same as town A	Same as town A
Height	An empirically derived rate estimated from CDC’s growth charts (12)	Same as town A	Same as town A
Weight	Calculated from height	Same as town A	Same as town A
BMI	BMI based on distribution analysis of NHANES 2003–2006 data to represent population average of childhood overweight/obesity	Distribution for town B is shifted 0.5 BMI units to the right (ie, greater BMI) of distribution for town A	Generated from CDC growth charts to represent “ideal” or “fitter-than-current-population-average” distribution
RMR	Determined according to Schofield equations, which estimate RMR from height (*H*), weight (*W*), age, and sex: • Boys aged 3–10 y, 19.59*W* + 1.303*H* + 414.9; boys aged 10–18 y, 16.25*W* + 1.372*H* + 515.5 • Girls aged 3–10 y, 16.969*W* + 1.618*H* + 371.2; girls aged 10–18 y, 8.365*W* + 4.65*H* + 200.0	Same as town A	Same as town A
**Agent behavior**			
Daily physical activity (energy expenditure)	Expressed in combinations of METs and duration by time of day (ie, before school, during school, and after school). Estimates derived from NHANES 2003–2006 accelerometer data.	Lower levels than those in town A: No physical education during school but some physical activity after school	Same as town A during school; more than town A after school
Daily dietary intake (energy intake)	Town A agents consume a multiple of their RMR daily; normal distribution derived from NHANES 2003–2006 data.	Same as town A	Multiplier lower than town A (by 0.01)
Sleep	10 hours per night, according to National Sleep Foundation (21) and other research (22)	Same as town A	Same as town A
Movement of agents from home to school and back on each weekday	15% of agents stop in the community to attend an after-school program on their way home from school (23)	Same as town A	Same as town A
**Time segment**			
School hours	8:00 am to 3:00 pm; assumption based on extant literature	Same as town A	Same as town A
Home hours	Midnight to 7:59 am; 7:00 pm to 11:59 pm; assumption based on extant literature	Same as town A	Same as town A
Community (after-school program)	3:01 pm to 6:59 pm; assumption based on extant literature	Same as town A	Same as town A
**Environment**			
No. of homes in town	56 homes; assumption based on modeling goals	Same as town A	Same as town A
No. of schools in town	12 schools; assumption based on modeling goals	Same as town A	Same as town A
No. of communities in town	1 community; assumption based on modeling goals	Same as town A	Same as town A
**Intervention – Active Schools Acceleration Project**			
Dose: amount of moderate to vigorous physical activity delivered by program	Based on ChildObesity180 observational data: • Program 1: 40 min @ 2.99 METs • Program 2: 10 min @ 2.62 METs • Program 3: 30 min @ 4.96 METs	Same as town A	Same as town A
Retention	Based on ChildObesity180 observational data and gray literature describing programs: • Program 1: 80% • Program 2: 90% • Program 3: 80%	Same as town A	Same as town A
Reach: the percentage of the student population who received the program	Based on ChildObesity180 observational data and gray literature describing programs: • Program 1: 10% • Program 2: 90% • Program 3: 75%	Same as town A	Same as town A
**Intervention –Healthy Kids Out of School Time**			
Dose	Based on ChildObesity180 observational data: Drink Right: decrease 60kcal; Move More: increase 15 min @ 4.5 METs; Snack Smart: decrease 68.25 kcals	Same as town A	Same as town A
Retention	80%; assumption	Same as town A	Same as town A
Reach	15%; assumption	Same as town A	Same as town A

Abbreviations: BMI, body mass index; CDC, Centers for Disease Control and Prevention; MET, metabolic equivalent of tasks; NHANES, National Health and Nutrition Examination Survey; RMR, resting metabolic rate.

a There are various definitions of agent-based modeling, but from a practical standpoint, an agent-based model is an individual-based approach to computer modeling in which agents (in this model, children aged 6–12), environments (in this model, the children’s community, or town), and the interventions (in this model, 2 obesity-prevention programs) are assigned properties; a computer simulation is run (in this model, agents move, eat, and exercise); and outcomes are generated (in this model, changes in BMI).

b The 2 interventions are the Active Schools Acceleration Project and Healthy Kids Out of School Time, both implemented by Tufts University–based ChildObesity180.

c Stylized towns represent potential “real world” communities in which ChildObesity180 interventions will be implemented.

For town A and town B, the BMI median was computed from NHANES 2003–2006 data (17,18). For town C, the BMI median was equal to the median defined in CDC’s growth charts. The BMI distribution for town A represented the average US community according to current childhood obesity rates (approximately 15% of children are obese). The BMI distribution in town B shifted toward the right (ie, greater BMI) of the town A median by 0.5 units. Town C was stylized to represent a BMI distribution before the rise of the US childhood obesity epidemic.

The NHANES 2003–2006 data sets were chosen for our model because they include objective measures of physical activity, 24-hour dietary recall data, and data on measured height and weight for children aged 6 to 12. The data sets also allowed for an empirically derived BMI distribution of the US population. To our knowledge, no other publicly available, nationally representative survey includes all of these features, not even the more recent cycles of NHANES data. We also chose to use baseline data from the same data set to the extent possible rather than merging data from different sources.

In addition to estimating the BMI distribution across town types, we defined 2 key behaviors: physical activity and dietary intake. Physical activity was expressed in combinations of metabolic equivalent of tasks (METs) and duration by time of day (ie, before school, during school, and after school). Town A physical activity estimates were derived from NHANES 2003–2006 accelerometer data. To obtain data in the form required for the model — estimates of moderate to vigorous physical activity (MVPA) by time of day — a novel methodology had to be developed (E.H. et al, unpublished data, 2015). In town B, we assumed that no physical education took place in school but some physical activity took place after school. In town C, we assumed that agents engaged in the same amount of physical activity as town A during school but slightly more physical activity after school. Using the Youth Compendium of Physical Activity, we defined MVPA as a MET value of 6.0 (19). Sleep (10-hour duration) was assigned a MET value of 1.0. Sleep duration was estimated from national recommendations on healthy sleep for children (20) and other research (21) because the NHANES data sets did not allow for this estimation. Time spent in sedentary activity was assigned a MET value of 1.5.

The model simulated each agent’s physical activity at minute increments during 24 hours and then converted these estimates into time-weighted average physical activity levels (PALs). The agent’s total energy expenditure (TEE) for that day equals its RMR multiplied by its PAL. For dietary intake, we assumed that town A agents consumed a multiple of their RMR daily and the data had a normal distribution derived from NHANES data. Dietary intake in town B was the same as that in town A, but in town C, the RMR multiplier was lower (by 0.01), reflecting healthier eating habits.

### Step 3. Characterize agent movement between home, school, and community

Next, the model was designed to follow this population of simulated children across 3 contexts: home, school, and community. In each community, a proportion of agents attend an after-school program; the proportion was based on national attendance at after-school programs, approximately 15% of the US child population (22). The model assumed the same distribution of after-school program attendance across town types because we could not find a data source that would explain whether this parameter would differ by town type. The model simulated energy intake and energy expenditure for each agent on each day in each context.

**Time.** Time was divided into 1-hour segments (or “ticks”). Agents moved between contexts according to time. At 08:00 all agents move from home to school. At 15:00 all agents leave school and move home or to community. At 19:00 all agents who are not already at home return home. Energy intake and expenditure were recorded hourly and then summed for the day at midnight to produce a calorie-gap estimate. The model ran for a period of 1 calendar year (365 days).

**Environment.** The environment consisted of 56 homes, 12 schools, and 3 community locations. The average elementary school has 400 students. These values were stylized identically for each town type; varying them would have no effect on the underlying dynamics of the model.

### Step 4. Characterize environmental change (intervention exposure)

The next step was to define and operationalize the interventions (ASAP and HKOS) to which the simulated agents would be exposed.

**ASAP.** Data were collected in spring 2013 by direct observation of physical activity performed by 100 children attending 6 ASAP elementary schools in California, New York, and Massachusetts (unpublished data, ChildObesity180, 2013). Dose was defined as the amount of MVPA delivered by each ASAP program. The stepwise process for estimating dose was the following:

Step 4a: Estimate RMR using the Schofield equation.
Step 4b. Estimate intervention energy cost (eg, time-weighted MET level) for each program.
Step 4c. Estimate energy expenditure from RMR and intervention data using inputs from Step 4a and 4b by sex, age, day, and program.

Reach was defined as the percentage of the student population who received the program. Using our observational data and publicly available program evaluation reports, we estimated that the classroom-based ASAP program (Program 2) would reach 90% of the student population, the before-school ASAP program (Program 1) would reach 10%, and Program 3 would reach 75%. Retention was defined as the percentage of the student population who remained in the program at the end of the year. We estimated that Program 1 and Program 2 would each retain 80% of the student population and Program 3 would retain 90%.

**HKOS.** We assumed that the Drink Right component would result in the intake of 60 fewer calories because agents would choose water over a sugar-sweetened beverage (23). For Snack Smart, we assumed that agents would consume 68.25 fewer kcals because they would substitute a standard cup serving of fruit for a typical energy-dense salty or sweet single-serve snack offered in an after-school program environment; this exchange was extrapolated from ChildObesity180 HKOS survey data (24).

The change in MVPA attributed to HKOS’s Move More component was 4.5 METs, a standard cut-point for characterizing MVPA (19). This cut-point was different from the cut-point used in ASAP. Based on the observational data, we knew the types of activities that were performed during ASAP (eg, Program 3 involved walking or running or both), so those activities could be modeled directly. With Move More, the physical activities of the different after-school programs (eg, sports practice vs scout meeting) varied; therefore a standard 4.5 MET definition was assumed. The model assumed 80% retention for HKOS. The Table provides a summary of the ABM properties, data inputs, and assumptions described in Steps 1–4.

### Step 5. Model implementation, refinement, and testing

**Figure 2:**
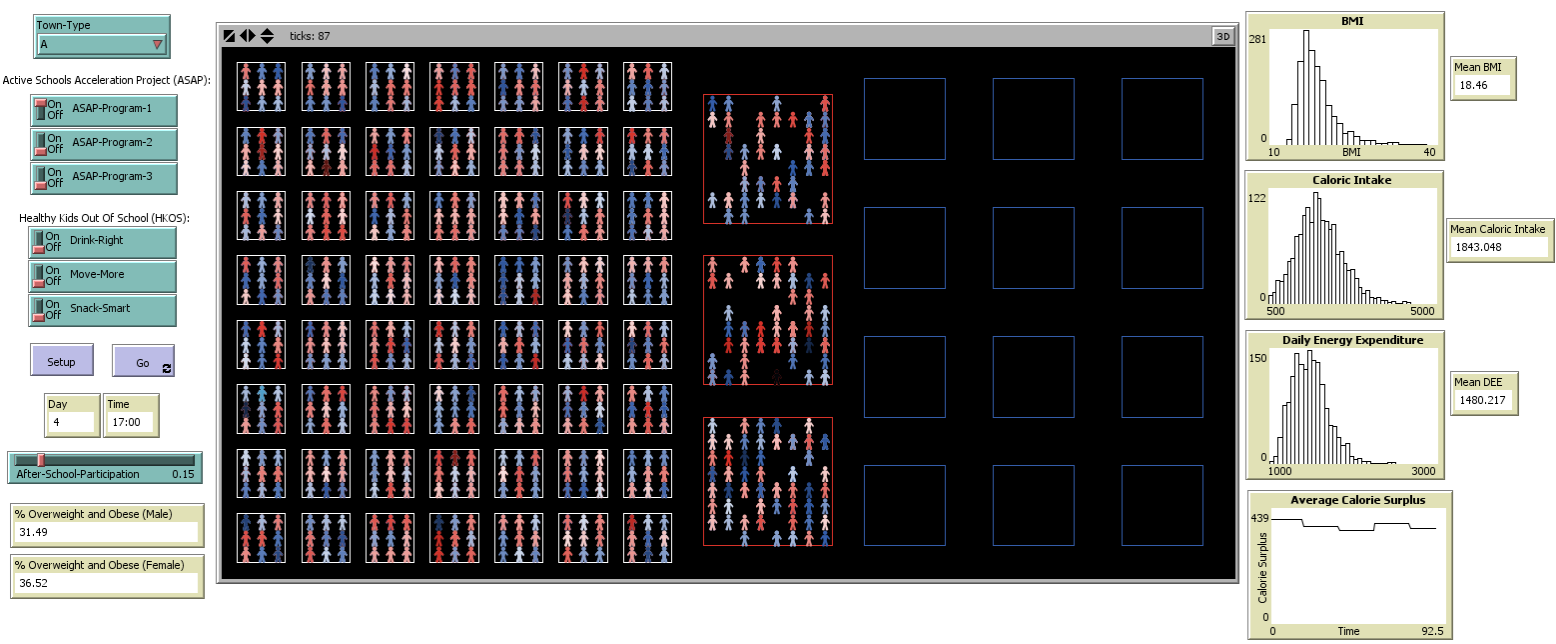
Overview of the agent-based modeling Netlogo interface. Each component of the model was programmed by the modeling team. Fields in the left column indicate each intervention or intervention component. For Town-Type (top of left column), users can select one of 3 town types (town A, town B, or town C); here town type A is selected. Moving down the column, for the Active Schools Acceleration Project (ASAP), each of 3 programs (Program 1, Program 2, and Program 3) is represented by an on/off switch; each program can be turned on or off independently of one another. For Healthy Kids Out of School (HKOS), each of 3 programs (Drink Right, Move More, and Snack Smart) is represented by an on/off switch; each program could be turned on or off independently of one another, but generally HKOS is treated as 1 intervention with all components turned on. The setup button initializes the simulation, creating agents according to assigned properties. The “go” button instructs the agents to carry out their behaviors. Two fields display outputs for the day and time reached by the simulation. A sliding scale for after-school participation characterizes the proportion of children who participate in the after-school program. At the bottom of the column, 2 fields show outputs of the percentage of children who are overweight and obese, by sex. Users select the “setup” then “go” buttons to allow agents to move, eat, and exercise in real time (illustrated in the center screen). The right column displays changes in agent or town properties (mean BMI, mean caloric intake, mean daily energy expenditure, and average calorie surplus) over time. Abbreviations: BMI, body mass index; DEE, daily energy expenditure.

The ABM was constructed using NetLogo 5.1.0 (https://ccl.northwestern.edu/netlogo/5.1.0), a multi-agent programmable modeling environment (Figure 2).

We have not yet completed Step 5. Completion will involve following best practices (25) for testing the model (using outcome data from the ChildObesity180 interventions, which are not yet available), refining assumptions and parameters iteratively, and conducting sensitivity analyses to establish the dependence of results on each assumption made. For instance, when we test the model, we will use data from ASAP to seed the initial distributions instead of generating stylized distributions. Other testing will involve using the intervention outcome data to determine how well the model predicts BMI change. Model refinement will include altering assumptions of the environment (eg, 365-day calendar vs an academic calendar), agent properties (eg, differences in behavioral patterns between weekdays and weekends), and intervention characteristics (eg, reach and retention estimates). Sensitivity analysis will systematically co-vary assumptions and parameters to establish a comprehensive portrait of model behavior.

## Conclusion

A model-based approach to intervention evaluation and design offers several benefits. First, it forces the researcher to be explicit about model assumptions. The model design process described in this article makes clear all the steps required to develop the model’s assumptions and the decision points at which the researcher must accept those assumptions or refine them. Second, knowledge from multiple sources can be leveraged. Our model builds on studies of RMR; population-based, public-use survey data; and primary source data. A quantitative model helps to consolidate this wide array of information to make it useful for prediction and evaluation of intervention outcomes. Third, models can be useful tools for communication: model assumptions must be formalized, and the implications of those assumptions must follow logically. As of this writing, no standard protocol exists for describing system-science simulation models, which can make them difficult to understand and duplicate. It is therefore important for the model builder to communicate clearly how the model was constructed and implemented.

The modeling effort outlined here stopped short of testing explanatory or predictive power; nonetheless, the modeling process uncovered important gaps in data and sparked new analysis to make the best use of existing data. The iteration between model refinement, data collection, and analysis can be a key contribution of formal models in the context of public health. 
